# Association between consumption of fruits and vegetables with suicidal ideation

**DOI:** 10.1017/S1368980021004687

**Published:** 2022-05

**Authors:** In Cheol Hwang, Seulggie Choi

**Affiliations:** 1 Department of Family Medicine, Gil Medical Center, Gachon University College of Medicine, 1198 Guwol-Dong, Namdong-Gu, Incheon, 405-760, South Korea; 2 Department of Biomedical Sciences, Seoul National University College of Medicine, Seoul, South Korea

**Keywords:** Cross-sectional studies, Fruit, Suicidal ideation, Vegetables

## Abstract

**Objective:**

To investigate the association between fruit and vegetable (F&V) intake with suicidal ideation.

**Design:**

Cross-sectional study using a Korean Community Health Survey.

**Setting:**

F&V consumers were defined as individuals who had consumed fruits or vegetables more than once per day. Multivariable logistic regression models were used to identify factors associated with suicidal ideation including F&V consumption and to estimate the prevalence of having suicidal ideation after consideration of potential confounders.

**Participants:**

221 081 Korea adults (nationally representative).

**Results:**

Approximately 55 % of participants were F&V consumers. They were more likely to be young, be women, attain high educational levels, be married and be healthier physically and psychologically than the F&V non-consumers. Non-consumers had an increased risk for suicidal ideation than consumers even when potential confounders were considered, and this trend was more remarkable with vegetable intake.

**Conclusion:**

F&V intake is associated with low risk for suicidal ideation.

Inarguably, suicide is a worldwide critical public health issue^(1)^. Each suicide has profound and lasting impacts on their families, friends and colleagues, not to mention large socio-economic costs. Identifying risk factors for suicide is imperative to improving our understanding of suicidal behaviours and ultimately preventing suicide. Previous studies have reported psychological, sociological, biological and environmental risk factors for suicide^(2)^. However, relatively few studies have investigated the links to dietary patterns with suicide risk.

There has been a growing body of literature on nutritional epidemiology with regards to emotional health^(3)^, with several potential mechanisms such as neuroinflammation, neurogenesis and synaptic plasticity etc^(4)^. Among them, a link between fruit and vegetable (F&V) consumption and psychological health has been suggested. Overall, high intake of F&V may promote higher levels of optimism and self-efficacy, reduce the level of psychological distress and protect against depressive symptoms^(5)^. An inverse association between depression, the most important risk factor for suicide, and dietary patterns characterised by high F&V intake has been documented^(6)^. Furthermore, higher consumption of F&V has been associated with decreased risk of various non-communicable diseases, which in turn may lead to better mental health^(7)^. However, to our knowledge, there is a paucity of research that examines whether the consumption of F&V is associated with suicidal behaviours^(5)^. Therefore, this study aimed to investigate the association between F&V consumption and suicidal ideation using data from a large-scale, population-based study in Korea.

## Methods

### Design and participants

**Fig. 1:**
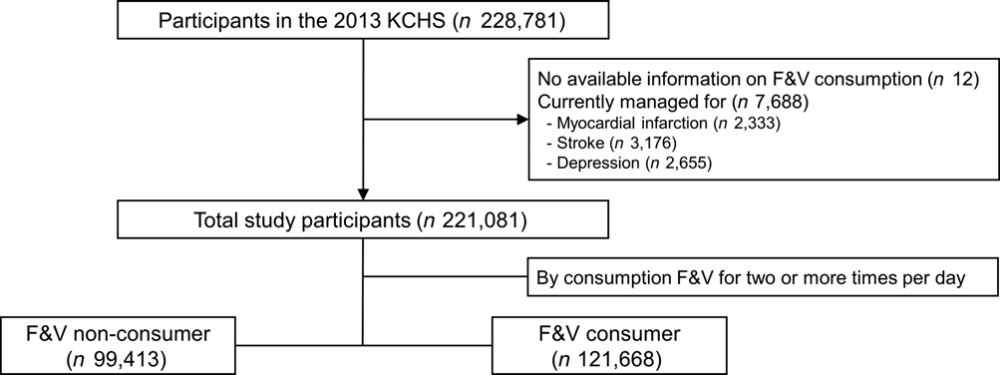
Schematic of participant selection. KCHS, Korea community health survey; F&V, fruit and vegetable

We used the 2013 Korea Community Health Survey (KCHS) database, which is a nationwide representative data using a multistage sampling design and has been annually performed by the Korea Centres for Disease Control and Prevention since 2008. In KCHS, a trained investigator visits the households and conducts a face-to-face interview for adults aged 19 years or older. The KCHS database contained various information to plan, implement, monitor and evaluate community health. We identified 228 769 individuals who had available information on consumption of F&V. After excluding patients with currently managed myocardial infarction, stroke and depression, 221 081 individuals were included in the final analysis (Fig. 1). More information about the KCHS database is found in detail elsewhere^(8)^.

### Main variables

Suicidal ideation was determined by the question, ‘Have you ever thought of suicide during the past year?’ Suicidal ideation was determined by the question, ‘Have you ever thought of suicide during the past year?’ F&V intake was assessed by the question, ‘How frequently have you consumed fruits or vegetables during the past month?’ Response options included ‘none,’ ‘less than one time per day,’ ‘one time per day,’ ‘two times per day’ or ‘three times or more per day.’ F&V consumer was defined as people who had consumed fruits or vegetables for two or more times per day.

### Covariates

Information on and/or its categorisation of covariates were followings: low economic status, covered by the Medicaid; low education attainment, middle school or less; unmarried included divorce, separation by any reasons and single; current smoker, smoked cigarettes at the time of interview; frequent drinker, drink more than 2 d/week; regular exercise, weekly physical activity of moderate-intensity activity more than five times or vigorous activity more than three times; obesity, BMI ≥ 25 kg/m^2^; hypertension and type 2 diabetes by a physician-diagnosed disease history; overall well-being score by a single 10-point scale; subjective health status by a 5 point Likert scale from ‘very good’ to ‘very bad’; stressful, responded question ‘how much stress do you have in your daily life?’ as ‘moderate’ or ‘severe’; and depressed, answered ‘yes’ to the question ‘have you ever felt sadness or despair continuously for more than 2 weeks during the past year?’

### Statistical analysis

Characteristics of participants by F&V consumption were presented as mean ± SD or number (percentage), and the differences between groups were tested by the *t*-test or *χ* ^2^ test. A backward stepwise multivariate regression model was used to identify factors associated with having suicidal ideation. To examine the dose–response effect, we performed multivariate logistic analyses calculating OR and 95 % CI of having suicidal ideation after consideration of potential confounders. All analyses were carried out using STATA se 9.2 (Stata Corp.), and results with *P* < 0·05 were considered statistically significant.

## Results

Table 1 shows the differences in characteristics of participants by F&V consumption. F&V consumers were more likely to be young, be women, less poor, have high educational attainment and be married. F&V consumers were also healthier than non-consumers: F&V consumers smoked or drank less, exercised more, were less obese, had less hypertension or type 2 diabetes, had higher well-being scores, and were less likely to report poor health. In mental health, F&V consumer were less stressful, less depressive and had less have suicidal ideation (all of *P*-values < 0·001).

**Table 1:** Characteristics of participants by consumption of vegetables or fruits

	Non-consumer (*n* 99 413)		Consumer (*n* 121 668)		*P*-value
	*n*	%	*n*	%	
Demographics					
Age, years					
Mean	52·7		50·6		<0·001
SD	18·3			15·8	
Women	50 043	50·3	71 826	59·0	<0·001
Coverage for low income	4292	4·3	2107	1·7	<0·001
Low education*	42 984	43·3	38 286	31·5	<0·001
Unmarried†	20 471	25·0	16 112	15·3	<0·001
Heath-related habits					
Current smoking	25 095	25·2	20 043	16·5	<0·001
Frequent drinking	24 387	24·5	23 491	19·3	<0·001
Regular exercise	21 866	22·0	30 977	25·5	<0·001
Comorbidities					
Obesity‡	22 823	24·5	27 518	23·2	<0·001
Hypertension	25 285	25·4	26 046	21·4	<0·001
Type 2 diabetes	9317	9·4	9776	8·0	<0·001
Overall health condition					
Well-being score§					
Mean	6·34		6·89		<0·001
SD	1·85		1·73		
Health status‖, bad	24 246	24·4	19 516	16·0	<0·001
Mental health					
Stressful¶	27 616	27·8	27 989	23·0	<0·001
Depressive**	5635	5·7	6088	5·0	<0·001
Suicidal ideation	11 480	11·6	9298	7·6	<0·001

* Middle school or lower.

† Including divorce, separation by any reason, and single.

‡ Body mass index ≥25 kg/m^2^.

§ By a single 10-point scale.

‖ By a 5-point Likert scale from ‘very good’ to ‘very bad’.

¶ By response to the question ‘how much stress do you have in your daily life?’ As ‘moderate’ or ‘severe’.

** By response to the question ‘have you ever felt sadness or despair continuously for more than 2 weeks during the past year?’ As ‘yes’.

Data are presented as means ± SD or numbers (percentages).

*P*-values were derived from the independent *t* test or the *χ* ^2^ test.

Table 2 identifies factors associated with suicidal ideation: various characteristics such as old age, women, low socio-economic status, unhealthy habits (current smoking, frequent drinking), type 2 diabetes, low well-being score, subjective health as bad, stressful and depressive mood remained significant. F&V non-consumers had 15 % increased odds for having suicidal ideation compared with F&V consumers (*P* < 0·001).

**Table 2:** Factors associated with suicidal ideation, including consumption of vegetables and fruits

	Crude OR	95 % CI	*P*-value	Adjusted OR*	95 % CI	*P*-value
Non-consumer of F&V	1·56	1·52, 1·61	<0·001	1·15	1·11, 1·20	<0·001
Old age, per 1 year	1·02	1·02, 1·03	<0·001	1·01	1·01, 1·01	<0·001
Women	1·81	1·76, 1·87	<0·001	1·61	1·53, 1·69	<0·001
Low income	3·36	3·17, 3·57	<0·001	1·31	1·20, 1·42	<0·001
Low level of education†	2·34	2·27, 2·41	<0·001	1·12	1·06, 1·17	<0·001
Unmarried‡	2·37	2·29, 2·45	<0·001	1·20	1·15, 1·25	<0·001
Current smoking	0·93	0·90, 0·97	<0·001	1·15	1·08, 1·21	<0·001
Frequent drinking	0·89	0·86, 0·92	<0·001	1·16	1·10, 1·21	<0·001
Type 2 diabetes	1·76	1·68, 1·84	<0·001	1·07	1·01, 1·13	0·018
Low well-being score, per 1-point	1·64	1·61, 1·64	<0·001	1·33	1·32, 1·33	<0·001
Subjective health as bad	3·96	3·85, 4·08	<0·001	1·53	1·46, 1·60	<0·001
Stressful	4·74	4·61, 4·89	<0·001	2·84	2·74, 2·96	<0·001
Depressive	14·54	13·97, 15·13	<0·001	7·82	7·44, 8·23	<0·001

* From the stepwise multivariate regression model adjusted for demographics (age, sex, economic status, educational attainment, marital status), health-related habits (current smoking, frequent drinking, regular exercise), comorbidities (obesity, hypertension, type 2 diabetes), overall health condition (well-being score, subjective health status), mental health (stress, depressive mood) and consumption of vegetables and fruits.

† Middle school or lower.

‡ Including divorce, separation by any reason and single.

**Fig. 2:**
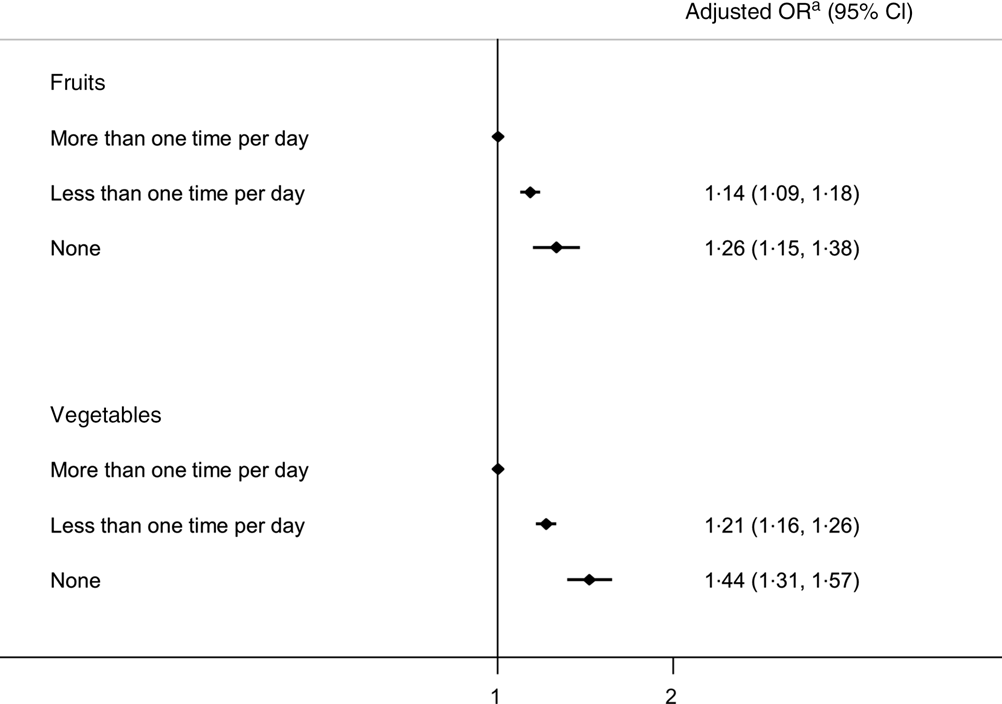
Suicidal ideation by the consumption of fruits and vegetables (both *P*_trend_ < 0·001). ^a^For demographics (age, sex, economic status, educational attainment, marital status), health-related habits (current smoking, frequent drinking, regular exercise), comorbidities (obesity, hypertension, type 2 diabetes), overall health condition (well-being score, subjective health status) and mental health (stress, depressive mood)

Figure 2 depicts the OR of having suicidal ideation by the consumption of fruits or vegetables, respectively. To examine the dose-responsive effect, we categorised participants into three groups (consumes fruit or vegetable more than 1 time per day, less than 1 time per day, and none) based on the distribution of current sample. Each consumption of fruit or vegetable was negatively associated with having suicidal ideation in a dose-dependent manner (OR 1·14; 95 % CI (1·09, 1·18)) and 1·26 (95 % CI (1·15, 1·38)) for fruit consumption; 1·21 (95 % CI (1·16, 1·26)) and 1·44 (95 % CI (1·31, 1·57)) for vegetable consumption), and the slope of association was steeper with vegetable consumption compared to fruit consumption.

## Discussion

Given the clinical trial showing the effectiveness of dietary supplements on suicidal behaviour and their well-beings^(9)^, exploring the relationship between diet and suicide like our study has implications for the prevention of suicide and for the development of nutrient intervention. By using unique data from a national survey, this study describes the likelihood of having suicidal ideation according to F&V intake. People who eat vegetables or fruits more than one time per day were at 15 % lower odds for having suicidal ideation compared to those who did not. The trend was more pronounced in vegetable consumption than in fruit consumption. The inverse association reported in this study between F&V intake and suicide is in line with previous findings^(10,11)^. Additionally, stronger association of vegetables consumption with suicidal ideation is consistent with the results of a recent meta-analysis^(12)^.

Only a handful of studies have examined the relationship between F&V intake and suicide. The data (*n* 6803) from the National Health and Nutrition Examination Survey suggest that F&V consumption was significantly lower in people with suicide attempts^(11)^. Another Japanese prospective study reported that a prudent diet including high intake of F&V was associated with a decreased risk of suicide death^(10)^. This was the largest study to date examining the relationship between separately assessed intake of F&V with suicidal ideation. The participants of KCHS were randomly selected from the community-dwelling population. Therefore, our results may be generalisable to adults in Korea. The current study also considered several potential confounders which might have otherwise influenced the results, such as mental illness and health-related behaviours at the time of dietary assessment^(5,13,14)^.

The mechanisms linking F&V intake and suicidal behaviours are still unknown, and they are not explained by any one biological pathway^(15)^. Several nutrients being rich in F&V (e.g. folate^(16)^, fibre^(17)^, C vitamin^(18)^, B vitamins^(19)^, carotenoids^(20)^, and polyphenols^(21)^) or their combination may have a beneficial effect on reducing suicide risk. In particular, polyphenols have been reported to be inversely associated with lower depressive symptoms^(22,23)^. Folate is also hypothesised to protect brain functions by reducing levels of homocysteine, which exerts neurotoxic effects^(24)^. Oxidative stress plays a significant role in mood disorders and the potential health benefits of F&V have been largely attributed to the antioxidant capacity of the constituents^(25,26)^. The gut–brain axis supports another potential mechanism: it links emotional centres of the brain with peripheral intestinal functions^(27,28)^. More nutritional data, such as micro- and macronutrients or polyphenols in fruit or vegetable, would be helpful to better speculate the mechanisms.

The major limitation of this study stems from the cross-sectional design and thus we could not determine the direction of the association between F&V intake and suicide ideation. Unhealthy diet and suicidal behaviour may also co-occur without being causally related to one another. Longitudinally repeated assessment of diet and suicidal behaviours would likely provide more evidence. Second, outcome assessment in this study was self-reported and was not based on a validated questionnaire. The self-reported response may introduce social desirability bias and it may have been difficult to recall single elements of a whole dietary pattern^(29)^. However, previous studies have demonstrated the validities of health-risk behaviour assessment via self-reported questionnaires despite the influence of cognitive and situational factors^(30)^. Third, additional consideration of other potential confounders such as food preference, family history of suicidal behaviour or interpersonal relationships is desirable.

Despite these limitations, this study indicates that low F&V intake is associated with suicidal ideation. Our findings suggest a need to improve dietary education and provide more nourishing diets to specific subpopulations. Additional studies in other health care systems or interventional studies exploring the effect of increasing F&V intake on suicidal behaviours are warranted.

## References

[1] Turecki G , Brent DA , Gunnell D et al. (2019) Suicide and suicide risk. Nat Rev Dis Primers 5, 74. 31649257 10.1038/s41572-019-0121-0

[2] Franklin JC , Ribeiro JD , Fox KR et al. (2017) Risk factors for suicidal thoughts and behaviors: a meta-analysis of 50 years of research. Psychol Bull 143, 187–232. 27841450 10.1037/bul0000084

[3] Marx W , Moseley G , Berk M et al. (2017) Nutritional psychiatry: the present state of the evidence. Proc Nutr Soc 76, 427–436. 28942748 10.1017/S0029665117002026

[4] Godos J , Currenti W , Angelino D et al. (2020) Diet and mental health: review of the recent updates on molecular mechanisms. Antioxidants 9, 346. 32340112 10.3390/antiox9040346 PMC7222344

[5] Glabska D , Guzek D , Groele B et al. (2020) Fruit and vegetable intake and mental health in adults: a systematic review. Nutrients 12, 115. 31906271 10.3390/nu12010115 PMC7019743

[6] Saghafian F , Malmir H , Saneei P et al. (2018) Fruit and vegetable consumption and risk of depression: accumulative evidence from an updated systematic review and meta-analysis of epidemiological studies. Br J Nutr 119, 1087–1101. 29759102 10.1017/S0007114518000697

[7] Angelino D , Godos J , Ghelfi F et al. (2019) Fruit and vegetable consumption and health outcomes: an umbrella review of observational studies. Int J Food Sci Nutr 70, 652–667. 30764679 10.1080/09637486.2019.1571021

[8] Kang YW , Ko YS , Kim YJ et al. (2015) Korea community health survey data profiles. Osong Public Health Res Perspect 6, 211–217. 26430619 10.1016/j.phrp.2015.05.003 PMC4551141

[9] Hallahan B , Hibbeln JR , Davis JM et al. (2007) *n*-3 Fatty acid supplementation in patients with recurrent self-harm. Single-centre double-blind randomised controlled trial. Br J Psychiatry 190, 118–122. 17267927 10.1192/bjp.bp.106.022707

[10] Nanri A , Mizoue T , Poudel-Tandukar K et al. (2013) Dietary patterns and suicide in Japanese adults: the Japan public health center-based prospective study. Br J Psychiatry 203, 422–427. 24115342 10.1192/bjp.bp.112.114793

[11] Li Y , Zhang J & McKeown RE (2009) Cross-sectional assessment of diet quality in individuals with a lifetime history of attempted suicide. Psychiatry Res 165, 111–119. 19046606 10.1016/j.psychres.2007.09.004

[12] Molendijk M , Molero P , Ortuno Sanchez-Pedreno F et al. (2018) Diet quality and depression risk: a systematic review and dose-response meta-analysis of prospective studies. J Affect Disord 226, 346–354. 29031185 10.1016/j.jad.2017.09.022

[13] Chi SH , Wang JY & Tsai AC (2016) Combined association of leisure-time physical activity and fruit and vegetable consumption with depressive symptoms in older Taiwanese: results of a national cohort study. Geriatr Gerontol Int 16, 244–251. 25657050 10.1111/ggi.12459

[14] Xiao Y , Romanelli M & Lindsey MA (2019) A latent class analysis of health lifestyles and suicidal behaviors among US adolescents. J Affect Disord 255, 116–126. 31150941 10.1016/j.jad.2019.05.031

[15] Marx W , Lane M , Hockey M et al. (2021) Diet and depression: exploring the biological mechanisms of action. Mol Psychiatry 26, 134–150. 33144709 10.1038/s41380-020-00925-x

[16] Gilbody S , Lightfoot T & Sheldon T (2007) Is low folate a risk factor for depression? A meta-analysis and exploration of heterogeneity. J Epidemiol Community Health 61, 631–637. 17568057 10.1136/jech.2006.050385 PMC2465760

[17] Xu H , Li S , Song X et al. (2018) Exploration of the association between dietary fiber intake and depressive symptoms in adults. Nutrition 54, 48–53. 29747090 10.1016/j.nut.2018.03.009

[18] Sahraian A , Ghanizadeh A & Kazemeini F (2015) Vitamin C as an adjuvant for treating major depressive disorder and suicidal behavior, a randomized placebo-controlled clinical trial. Trials 16, 94. 25873303 10.1186/s13063-015-0609-1 PMC4376513

[19] Mikkelsen K , Stojanovska L & Apostolopoulos V (2016) The effects of vitamin B in depression. Curr Med Chem 23, 4317–4337. 27655070 10.2174/0929867323666160920110810

[20] Milaneschi Y , Bandinelli S , Penninx BW et al. (2012) The relationship between plasma carotenoids and depressive symptoms in older persons. World J Biol Psychiatry 13, 588–598. 21929378 10.3109/15622975.2011.597876 PMC3360996

[21] Sureda A & Tejada S (2015) Polyphenols and depression: from chemistry to medicine. Curr Pharm Biotechnol 16, 259–264. 25601603 10.2174/1389201016666150118133313

[22] Godos J , Castellano S , Ray S et al. (2018) Dietary polyphenol intake and depression: results from the Mediterranean healthy eating, lifestyle and aging (MEAL) study. Molecules. 23, 999. 29695122 10.3390/molecules23050999 PMC6102571

[23] Chang SC , Cassidy A , Willett WC et al. (2016) Dietary flavonoid intake and risk of incident depression in midlife and older women. Am J Clin Nutr 104, 704–714. 27413131 10.3945/ajcn.115.124545 PMC4997290

[24] Bottiglieri T (2005) Homocysteine and folate metabolism in depression. Prog Neuropsychopharmacol Biol Psychiatry 29, 1103–1112. 16109454 10.1016/j.pnpbp.2005.06.021

[25] Logan AC (2006) Dietary fiber, mood, and behavior. Nutrition 22, 213–214. 16459235 10.1016/j.nut.2005.06.005

[26] Dragsted LO , Pedersen A , Hermetter A et al. (2004) The 6-a-day study: effects of fruit and vegetables on markers of oxidative stress and antioxidative defense in healthy nonsmokers. Am J Clin Nutr 79, 1060–1072. 15159237 10.1093/ajcn/79.6.1060

[27] Mena P & Bresciani L (2020) Dietary fibre modifies gut microbiota: what’s the role of (poly)phenols? Int J Food Sci Nutr 71, 783–784. 32993403 10.1080/09637486.2020.1826913

[28] van der Merwe M (2021) Gut microbiome changes induced by a diet rich in fruits and vegetables. Int J Food Sci Nutr 72, 665–669. 33960869 10.1080/09637486.2020.1852537

[29] Tapsell LC , Neale EP , Satija A et al. (2016) Foods, nutrients, and dietary patterns: interconnections and implications for dietary guidelines. Adv Nutr 7, 445–454. 27184272 10.3945/an.115.011718 PMC4863273

[30] Centers for Disease Control and Prevention, Brener ND , Kann L et al. (2013) Methodology of the youth risk behavior surveillance system–2013. MMWR Recomm Rep 62, 1–20. 15385915

